# Driving Rotational Circulation in a Microfluidic Chamber Using Dual Focused Surface-Acoustic-Wave Beams

**DOI:** 10.3390/mi16020140

**Published:** 2025-01-25

**Authors:** Jin-Chen Hsu, Kai-Li Liao

**Affiliations:** Department of Mechanical Engineering, National Yunlin University of Science and Technology, Douliu 64002, Taiwan; snesjacky94005@gmail.com

**Keywords:** focused surface acoustic wave, focused interdigital transducer, acoustic streaming, microparticle, microfluidics, acoustic chamber

## Abstract

In this paper, enhanced rotational circulation in a circular microfluidic chamber driven by dual focused surface-acoustic-wave (SAW) beams is presented. To characterize the resonant frequency and focusing effect, we simulate the focused SAW field excited by an arc-shaped interdigital transducer patterned on a 128°*Y*-cut lithium-niobate (LiNbO_3_) substrate using a finite element method. A full three-dimensional perturbation model of the combined system of the microfluidic chamber and the SAW device is conducted to obtain the acoustic pressure and acoustic streaming fields, which show rotational acoustic pressure and encircling streaming resulted in the chamber. Accordingly, the SAW acoustofluidic system is realized using microfabrication techniques and applied to perform acoustophoresis experiments on submicron particles suspending in the microfluidic chamber. The result verifies the rotational circulation motion of the streaming flow, which is attributed to enhanced angular momentum flux injection and Eckart streaming effect through the dual focused SAW beams. Our results should be of importance in driving particle circulation and enhancing mass transfer in chamber embedded microfluidic channels, which may have promising applications in accelerating bioparticle or cell reactions and fusion, enhancing biochemical and electrochemical sensing, and efficient microfluidic mixing.

## 1. Introduction

Surface acoustic wave (SAW) devices have been widely used in radio-frequency (RF) signal processing and bandpass filter applications, and thus have become the crucial basis of mobile communications [1,2,3]. The success of SAW devices has opened the way for recent development of the active microfluidics using acoustic fields, allowing it to demonstrate several important capabilities, including rapid mixing of fluids and manipulation of immersed particles [4,5,6]. SAW-based microfluidic technology utilizes the same method for SAW generation as the SAW devices used as filters and resonators, which is based on applying an RF signal to an interdigital transducer (IDT) patterned on a piezoelectric substrate. The time-varying redistribution of electrical charges causes continuous deformation of the substrate surface (i.e., the piezoelectric effect), thereby emitting megahertz (MHz) SAWs, which simultaneously produce longitudinal and transverse vibrations along the propagation of the waves. The working mechanism of SAW microfluidics is therefore based on the effective acoustic energy transferred from a SAW to the fluid on the substrate. Compared to other energy sources used in active microfluidics such as thermal, magnetic, and electric fields [7,8,9], the apparent advantages of SAW are large mechanical force, fast actuation, and simple electrode network [6,10]. Nowadays, the highly mature photolithography and etching technologies also make the fabrication of SAW devices relatively cost-effective. Although traditional SAW devices use different piezoelectric materials, most SAW-driven microfluidic devices prefer to use 128° rotated *Y*-cut single-crystal lithium niobate (128°*Y*-cut LiNbO_3_) substrates due to the relatively high electromechanical coupling coefficient and biocompatibility.

In microfluidic applications, SAW devices can be further classified into standing SAW (SSAW) and travelling SAW (TSAW) devices [11,12,13], where SSAW is the superposition of two counter-propagating TSAWs. SSAW devices have been developed for efficient focusing, sorting, and separation of cells or particles [14,15,16]. Acoustic radiation forces (ARF) on particles dispersed in a fluid by SSAW are much larger than the radiation forces exerted by TSAW at a given frequency and power input. TSAW, on the other hand, is concomitant with higher acoustic streaming flow velocity [17]. It is known that acoustic streaming flow (ASF) is particularly useful for acoustically driving fluid circulation. Two main types of ASF can typically be generated by SAWs, which are Rayleigh–Schlichting streaming and Eckart streaming [18,19]. Rayleigh–Schlichting streaming is formed by dissipating acoustic energy into the boundary layer along non-slip solid boundaries, thereby generating local vortices driven by a viscous boundary layer. These boundary-driven vortices are confined to a short distance from the boundaries (typically less than the acoustic wavelength) and consume significant acoustic power. Eckart streaming, on the other hand, is formed by the dissipation of acoustic energy into the bulk of a fluid, creating a spatially dispersed fluid jet that is propelled by an acoustic beam in the propagation direction of acoustic waves. Accordingly, Eckart streaming can be generated over a longer acoustic attenuation length. Acoustic streaming flows induced by TSAWs have proven to be particularly useful for particle sorting, fluid mixing, and liquid pumping inside microfluidic channels [20,21,22].

In this study, we employ focused TSAWs to drive strong rotational circulation in a circular microfluidic chamber. To enhance the delivery of angular momentum flux carried by the Eckart-type streaming into the fluids in the chamber, two misaligned focused IDTs are patterned on the surface of a 128°*Y*-cut LiNbO_3_ substrate to generate two offset focused TSAW beams. The microfluidic chamber provides an expanded circular space to accommodate the long-range Eckart streaming flows induced by the focused TSAWs. The strong rotational circulation of microfluid can be particularly useful for applications such as rapid micromixing, accelerating bioparticle or cell reactions and fusion, and enhancing continuous electrochemical sensing [23,24,25]. In this work, we aim to establish the design, implementation, and mechanisms of the SAW device for driving a strong rotational circulation in the microfluidic chamber.

## 2. Materials and Methods

### 2.1. Working Principle and Numerical Approach

**Figure 1:**
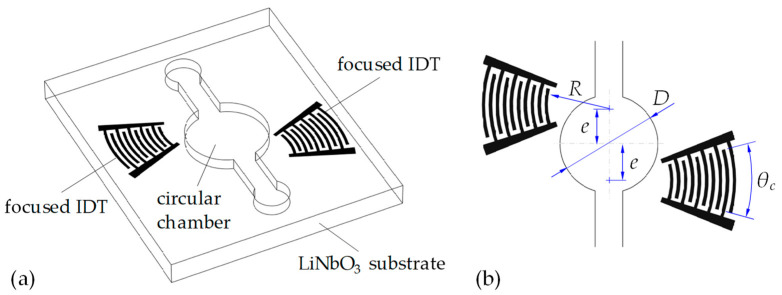
(**a**) Schematics of the device structure. A microfluidic chamber is actuated by two misaligned focused SAWs excited using the offset IDTs on a LiNbO_3_ substrate. (**b**) Geometrical parameters of the device, where *D* is the chamber diameter, *R* is the radius of the first electrode of the focused IDTs, *θ_c_* is the coverage angle of the focused IDTs, and *e* is the offset distance of the IDTs from the horizontal centerline of the circular chamber.

Figure 1 illustrates the structure of the device. In this design, a circular chamber of diameter *D* is symmetrically embedded in a closed microchannel of width *w*, which creates an expanded space in the channel for unrestrainedly driving the microfluidic circulation by TSAWs. The channel height is denoted as *h*. This circular chamber-embedded microchannel device is integrated on the surface of a 128°*Y*-cut LiNbO_3_ substrate of thickness *h*_LN_. On both sides of the circular chamber, two focused IDTs are patterned on the surface of the 128°*Y*-cut LiNbO_3_ substrate with an offset of *e* with respect to the horizontal centerline of the circular chamber. These offset focused IDTs are electrically actuated to excite two misaligned focused SAW beams that propagate in opposite directions, creating oppositely directed offset acoustic streaming flows in the chamber. Consequently, this spatially tailored acoustic streaming flows can drive strong rotational circulation of the contained fluid.

To proceed a rational design of the device, we adopt a finite-element (FE) approach combined with the acoustofluidic perturbation method and electro-elastodynamics [26,27]. The simulations using the FE analysis can provide three-dimensional (3D) full-wave information of the device. The overall system of the FE algorithm integrates the piezoelectric solid and chamber fluid domains of the device model with continuous physical conditions along their interface. In the piezoelectric solid domain, the mechanical displacement *u_j_* and electric displacement *D_i_* of elastic-wave propagation are governed by Cauchy’s equation and Gauss’s law, respectively [28]:

(1) $$\begin{array}{l} \partial_{i}T_{ij}=-\rho_{s}\omega^{2}u_{j},\\ \partial_{i}D_{i}=0, \end{array}$$

where *ρ*_s_ is the mass density of the solid, *ω* is the angular frequency, and *T_ij_* is the elastic stress tensor. The stress and electric displacement obey the piezoelectric constitutive laws [29]

(2) $$\begin{array}{l} T_{ij}=c_{ijkl}S_{kl}-e_{kij}E_{k},\\ D_{i}=e_{ikl}S_{kl}+\varepsilon_{0}\varepsilon_{ik}E_{k}, \end{array}$$

where *S_kl_* and *E_i_* are the elastic strain tensor and the electric field, respectively; *c_ijkl_* is the elastic stiffness, *e_kij_* is the piezoelectric coefficient; *ε_ik_* is the dielectric constant; and *ε*_0_ is the vacuum permittivity. The strain is related to the displacement by $S_{kl}=\left(\partial_{l}u_{k}+\partial_{k}u_{l}\right)/2$, and the electric field is related to the electric potential by $E_{i}=-\partial_{i}\varphi$.

The electrical excitation of focused SAWs can be modeled by imposing surface electric potentials *ϕ_c_* and *ϕ_g_* on the charged and grounded electrodes of the focused IDTs on the top surface of the LiNbO_3_ substrate as follows [26,27]:

(3) $$\left\{\begin{array}{c} \;\varphi_{c}=V_{0}\;e^{i\omega t},\;\\ \;\varphi_{g}=0. \end{array}\right.$$

Along the non-electrode surfaces of the piezoelectric substrate that are in contact with air, the stress-free and open-circuit boundary conditions are specified:

(4) $$\left\{\begin{array}{c} T_{ij}n_{j}=0,\\ D_{j}n_{j}=0, \end{array}\right.$$

where *n_j_* is the unit vector outwardly normal to the boundary.

In the fluid domain, the equations that govern the acoustic and streaming flow fields are generated by asymptotic expansion of the conservation laws of mass and momentum. The velocity *v*_1*j*_ and pressure *p*_1_ of the acoustic field are described by the first-order equations as follows [26,27,30]:

(5) $$\begin{array}{l} -i\omega \rho_{1}+\rho_{0}\partial_{j}v_{1j}=0,\\ -i\omega \rho_{0}v_{1j}=-\partial_{i}p_{1}\delta_{ij}+\eta \partial_{k}\partial_{k}v_{1j}+\left(\eta_{B}+\frac{1}{3}\eta \right)\partial_{j}\partial_{l}v_{1l}, \end{array}$$

where *ρ*_0_ and *ρ*_1_ are the undisturbed and first-order densities of the fluid, respectively, and *η* and *η_B_* are the shear and bulk viscosities, respectively. Here, the first-order fluctuations of the density and pressure can be related by the constitutive relation *ρ*_1_*c*_0_^2^ = *γp*_1_, where *c*_0_ and *γ* are the speed of sound and the specific heat capacity of the fluid. The acoustic streaming flow field can be described by the time-averaged second-order equations as follows [26,27,30]:

(6) $$\begin{array}{l} \langle \rho_{0}\partial_{j}v_{2j}\rangle =-\partial_{j}\langle \rho_{1}v_{1j}\rangle ,\\ \langle \rho_{1}{\dot{v}}_{1j}\rangle +\rho_{0}\langle v_{1k}\partial_{k}v_{1j}\rangle =-\partial_{i}\langle p_{2}\delta_{ij}\rangle +\eta \partial_{k}\partial_{k}\langle v_{2j}\rangle +\left(\eta_{B}+\frac{1}{3}\eta \right)\partial_{j}\partial_{l}\langle v_{2l}\rangle , \end{array}$$

where *v*_2*j*_ and *p*_2_ are the second-order velocity and pressure, respectively. In Equation (6), the source terms to actuate the streaming velocity *v*_2*j*_ result from nonlinear products of the first-order quantities.

The first- and second-order fields are obtained through a two-step solution procedure using commercial FE solver package COMSOL Multiphysics [31]. First, the acoustic field of the full-wave model can be solved using the equation system that couples Equations (1) and (5) together with the continuity of velocity and stress along the solid–fluid interface, which is given by [26]

(7) $$\left\{\begin{array}{c} i\omega \;u_{j}=v_{1j},\\ T_{ij}n_{j}=\left(-p_{1}\delta_{ij}+\eta \left(\partial_{i}v_{1j}+\partial_{j}v_{1i}\right)+\left(\eta_{B}-\frac{2}{3}\eta \right)\partial_{k}v_{1k}\delta_{ij}\right)\;n_{j}. \end{array}\right.$$

After the first-order acoustic field is obtained, the time-averaged second-order streaming field in the fluid domain can be solved accordingly using Equation (6) with the non-slip hard boundary condition.

### 2.2. Device Fabrication and Experimental Procedure

The designed acoustofluidic system consists of two device components, i.e., the SAW device and the microfluidic device. In the SAW device fabrication, a standard photolithographic technology was adopted [32,33]. The used piezoelectric substrate is a 128°*Y*-cut LiNbO_3_ wafer of 500-μm thickness. The fabrication process involves deposition of a thin Cu/Ti metal film on the LiNbO_3_ wafer, spin-coating of photoresist on the metal film, development of focused IDT pattern on the photoresist by ultraviolet exposure, and finally chemical wet etching of the metal film to form focused IDT electrodes.

The microfluidic device was fabricated using the replica molding method [34]. The monolithic master mold was created using a micro-milling process. After finishing the machining of the master mold, a mixture of PDMS base and curing agent (Sylgard 184, Dow Corning, Midland, MI, USA) at a ratio of 10:1 *w*/*w* was poured into the master mold to cast the microchannel structure by complying with a standard degassing and solidifying process. Finally, the PDMS replica of the chamber-embedded microchannel device was accurately combined with the SAW device through surface activation by oxygen plasma to form the SAW acoustofluidic system.

The SAWs at the frequency that is effective to be electrically excited in the fabricated device were pre-determined using a network analyzer (Agilent E5061B, Keysight, Rosebery, NSW, Australia). Subsequently, in acoustophoretic experiments, the microfluidic device was actuated by injecting liquid with suspended particles into the microchannel using a syringe pump. The RF voltage produced at the measured SAW frequencies by a power amplifier (75A250A, Amplified Research, Souderton, PA, USA) was applied to the focused IDTs to excite the dual focused SAW beams to generate the acoustophoretic forces on the particles. The resulting particle motion was recorded using a CMOS camera (E3ISPM, Sony, Tokyo, Japan) mounted on an inverted microscope (ECLIPSE Ti2-U, Nikon, Tokyo, Japan).

## 3. Results

### 3.1. Design and Excitation of the Focused SAWs

**Figure 2:**
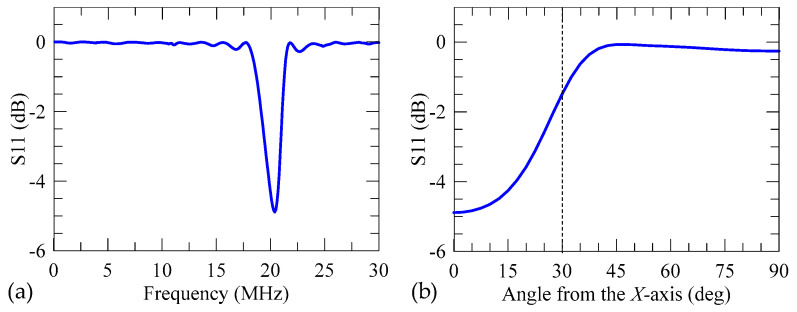
(**a**) Calculated S11 spectrum of 20-pair IDT of *p*_IDT_ = 200 μm on the 128°*Y*-cut LiNbO_3_ along the *X* direction using the 2D FE model, which shows a resonant frequency at 20.4 MHz. (**b**) Calculated S11 value when IDT of the same pitch is arranged long other directions at an angle *θ* to the *X* direction and excited at the frequency of 20.4 MHz, showing that the excitation efficiency of the IDT decreases as *θ* is increased. A threshold (the dashed line) is set at *θ* = 30° for the focused IDT design.

The main propagation direction of the focused SAW is along the *X* direction of the 128°*Y*-cut LiNbO_3_ wafer. To estimate the resonant frequency of an IDT with a pitch *p*_IDT_, we calculated the S11 spectrum of the SAW device using a simplified two-dimensional (2D) cross-sectional FE model developed in Ref. [26]. Using a 2D model relieves the heavy numerical burden of directly using a 3D model when calculating the S11 spectrum in the frequency domain. In our design, the pitch *p*_IDT_ = 200 μm and the number of electrode pairs *N_e_* = 20 were selected. The calculated S11 spectrum is shown in Figure 2a, where an obvious frequency dip occurs at 20.4 MHz. This frequency dip reveals that the IDT can effectively emit SAW when the applied electrical potential is at this frequency. Correspondingly, the numerical SAW velocity for this 128°*Y-X* LiNbO_3_ substrate is *c*_SAW_ = 4080 m/s. The associated displacement field excited at the dip frequency in the S11 spectrum exhibits a SAW mode. Based on this SAW frequency (denoted as *f*_SAW_), Figure 2b shows the calculated S11 value when IDT of the same pitch is excited along other directions at an angle *θ* to the main direction using the 2D model. In this calculation process, we aligned the desired propagation direction along the *x* direction of the 2D model by transforming the physical properties of 128°*Y*-cut LiNbO_3_. In Figure 2b, it is observed that the S11 value increases with the increase in the angle *θ*. This means that the excitation efficiency decreases as the angle *θ* increases. Excitation efficiency of the IDT at 20.4 MHz indicated by the S11 value tends to zero when *θ* is larger than 45°. Therefore, a coverage angle *θ_c_* = 60° (i.e., *θ* = ±30°) was adopted as our focused IDT design.

**Figure 3:**
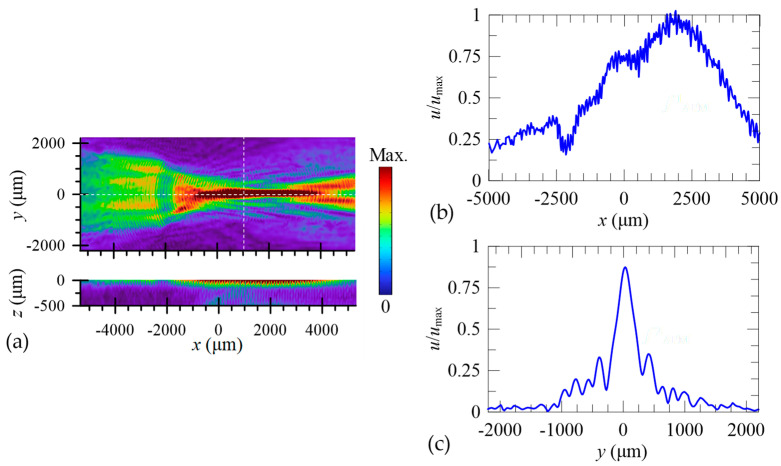
(**a**) Calculated focused SAW field at the frequency *f*_SAW_ = 20.4 MHz using a full 3D model, which shows a highly focused SAW beam achieved by the arc-shaped IDT. (**b**) Horizontal and (**c**) vertical distributions of the displacement amplitude on the top surface of the LiNbO_3_ substrate along the dashed lines in (**a**).

**Figure 4:**
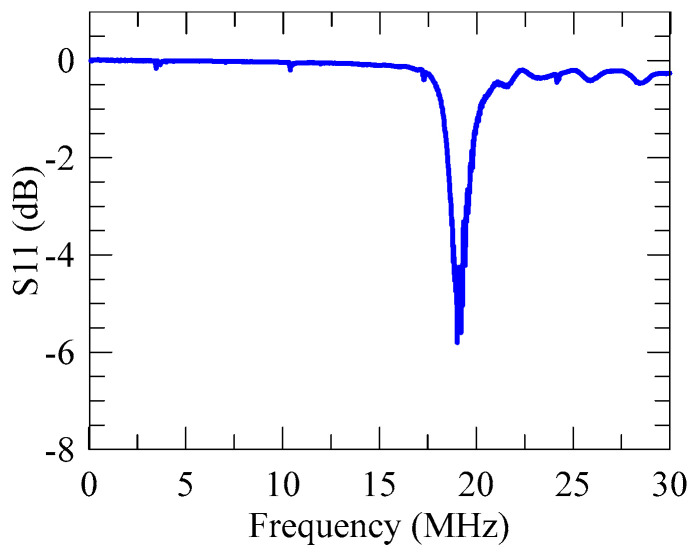
Measured S11 spectrum of the fabricated focused SAW device with *p*_IDT_ = 200 μm, *N_e_* = 20, and *θ_c_* = 60°. The measured resonant frequency occurs at 19.1 MHz.

Using an arc-shaped focused IDT with the chosen coverage angle, Figure 3a show the calculated focused SAW field at the frequency *f*_SAW_ = 20.4 MHz using a full 3D model. Outside the domain of interest, perfectly matched layer (PML) surrounding was utilized to absorb the out-going waves and prevent unwanted reflections by the domain boundary. The result demonstrates a highly focused SAW beam excited by the focused IDT of the designed radial pitch *p*_IDT_, where the displacements of focused field are localized near the top surface of the substrate. Figure 3b,c show the distributions of the displacement amplitude on the top surface of the LiNbO_3_ substrate along specific horizontal and vertical lines (the dashed lines in Figure 3a). The focused SAW beam exhibits a relatively long focal beam length and a narrow beam width. Intriguingly, the displacement amplitude field reaches its highest value at *x *= 2000 μm, which is 3600 μm from the first IDT electrode. In other words, the densest spot of displacement amplitudes occurs at a position 2000 μm to the right of the theoretical focal point at *x *= 0 μm (i.e., the arc center). Rather than considering this difference as a theoretical or numerical inaccuracy, we attribute the formation of the densest spot at *x *= 2000 μm to the secondary focusing effect due to the complex propagation and diffraction behavior of SAW in the 128°*Y*-cut LiNbO_3_ substrate. The mechanism behind the secondary focusing could be the piezoelectric anisotropy of the LiNbO_3_ substrate. The corresponding full width at half maximum (FWHM) value of the focused beam are 1.97*λ*_SAW_. The long focal beam length potentially benefits the generation of Eckart streaming flows through a longer spatial coupling of SAW and fluid. Figure 4 shows the measured S11 spectrum of the fabricated focused SAW device. The obtained dip frequency from the measurement is 19.1 MHz, which agrees well with the numerical value at 20.4 MHz. The deviation of numerical frequency from the experimental frequency is 6.8%. We attribute this frequency deviation is mainly due to the difference in physical properties of the 28°*Y*-cut LiNbO_3_ in the numerical model from the experimental material.

### 3.2. Acoustic Field and Streaming Velocity Field

Based on the simulated results of the SAW excitation and focusing in Section 3.1, a 3D device model with a water-filled circular chamber domain and a pair of misaligned focused IDTs was established to obtain the acoustic pressure and streaming fields using the equations presented in Section 2.1. The geometrical parameters of the designed device are summarized in Table 1.

**Figure 5:**
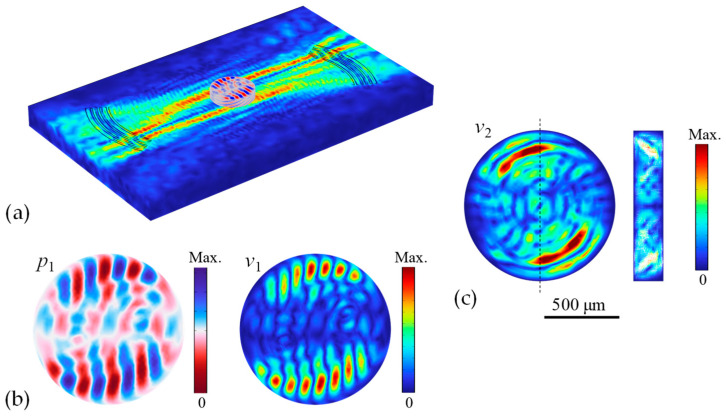
(**a**) Coupled acoustic-wave field of the SAW device and the microfluidic chamber, where the chamber diameter *D *= 1000 μm. (**b**) Top views of the acoustic pressure field and the associated first-order velocity field in the circular chamber. (**c**) Top view and cross-section view of the second-order streaming field in the chamber.

Figure 5 shows the calculated acoustic and streaming fields driven by the two focused SAW beams. The full acoustic-wave field shown in Figure 5a demonstrates the coupling interaction between the focused SAW beams and the water-filled circular chamber. It can be observed that the two offset focused SAW beams generate phase-distorted acoustic pressure that propagates rotationally around the chamber (also see the dynamic process in Supplementary Movie S1). A top view of the acoustic pressure field and the associated first-order velocity field in the circular chamber are shown in Figure 5b. This rotational acoustic pressure field contains a boosted nonzero angular momentum flux injected by the two focused beams to drive fluid motion. The first-order velocity field is also rotational; thus, the vibration of the fluid elements also propagates rotationally around the chamber (see the dynamic process in Supplementary Movie S2). Furthermore, these first-order fields actuate the second-order streaming field, as shown in Figure 5c. Strong streaming velocities yield near the top and bottom circumference edges of chamber where the focused SAW beams coupled directly to launch long-range Eckart streaming flow. This actuation can generate a strong encircling circulation of the fluid in the chamber. A cross-sectional view of the streaming velocity field (cut along the vertical centerline of the chamber) is shown on the right side. Several local rolling vortices caused by the bottom viscous boundary-driven Rayleigh–Schlichting streaming can be observed. In view of these first-order acoustic field and second-order streaming field, it is expected that the resulting acoustic forces can be valid to drive rotational circulation of fluid or suspended particles in this circular chamber.

To gauge the strength of the rotational circulation, we applied an RF voltage amplitude of 1.0 V on the focused IDTs in the numerical simulations to calculate the acoustic streaming velocity field. The results show that the amplitude of the focused SAWs attains 2.0 nm and the maximum acoustic streaming velocity in the microfluidic chamber achieves 18.52 mm/s (without considering any loss and attenuation). Obviously, this high acoustic streaming velocity is attributed to the high power intensity of the focused SAWs. In our previous study [21], the amplitude of plane-wave SAWs excited by conventional unfocused IDT using the same RF voltage was well below 0.5 nm. The achieved acoustic streaming velocities were on the order of 0.01–0.1 mm/s (where the radiation loss and attenuation of SAWs in the PDMS cover layer were included), which is much slower than those achieved in this study. Therefore, these quantitative numerical data suggest that the dual focused SAW beams can drive strong rotational circulation inside a circular chamber.

**Figure 6:**
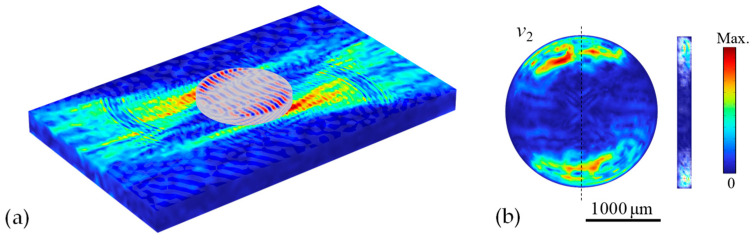
(**a**) Coupled acoustic-wave field of the SAW device and the microfluidic chamber, where the chamber diameter *D *= 2000 μm. (**b**) Top view and cross-section view of the second-order streaming field in the chamber.

To provide additional insights into the impact of chamber size, Figure 6 shows the simulated results of acoustic and streaming velocity fields for the case of a larger microfluidic chamber with a diameter *D *= 2000 μm. The dual focused SAW beams are launched to actuate the fluid from the top and bottom edges of the circular chamber (i.e., *e *= 1000 μm). The results show that the overall rotational circulation in the larger chamber is not as strong as that in the smaller chamber driven by the dual focused SAW beams. The larger chamber has a longer radius and a larger fluid volume, so the focused SAWs with a small focused beam width at the same power level have difficulty effectively driving the fluid near the central region of the chamber, which suggests that larger microfluidic chambers require higher SAW power to achieve the strong rotational circulation. When designing the microfluidic chamber, a crucial consideration is the SAW wavelength and the achieved focused beam width (typically 1–2 times the SAW wavelength) compared to the chamber dimensions. A chamber radius of 2–3 times the SAW wavelength would be a reasonable size for effective actuation. Therefore, we used the SAW wavelength of 200 μm for the circular chamber with a radius of 500 μm in this study. Moreover, since the presented device capable of enhanced circulation can benefit mass transfer rate, it is beneficial to facilitate efficient high-viscosity microfluidic mixing.

### 3.3. Rotational Circulation of Submicron-Particles

**Figure 7:**
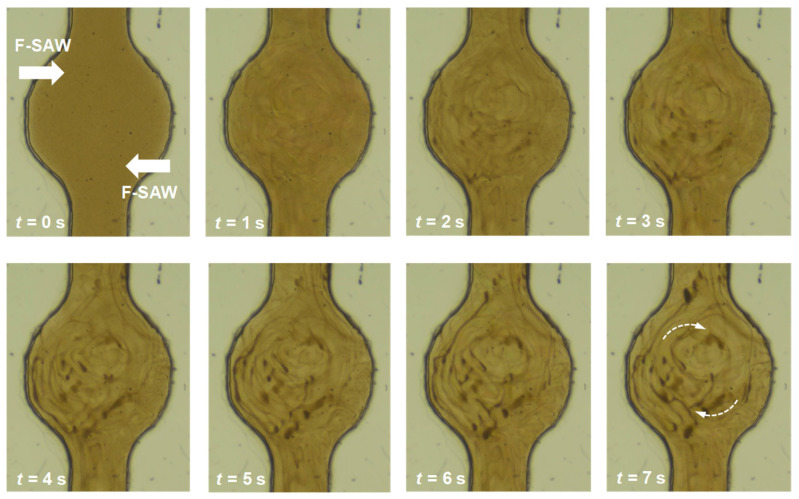
Time-evolution images of particle motion in the microfluidic chamber driven by the dual focused SAW beams, showing strong rotational circulation of the particle streams. The dashed arrows indicate the direction of circulation.

According to the resulting acoustic pressure and streaming fields induced by the dual focused SAWs investigated in Section 3.2, the acoustic radiation forces due to the rotational first-order acoustic field and Stokes drag force induced by the encircling second-order streaming field would drive particles suspended in the microfluidic chamber to rotate around the chamber. In this study, we focus on streaming-dominated acoustophoresis driven by the SAW device. This can be estimated using the acoustophoretic parameter *κ = πf*_ac_*d*_p_/*c*_f_ by satisfying *κ* < 1, where *d*_p_ is the particle diameter, *f*_ac_ is the acoustic frequency, and *c*_f_ is sound speed in the fluid [35]. Figure 7 shows the time-evolution images of particle motion in the chamber where strong rotational circulation of particle streams can be observed (also see Supplementary Movie S3). In this acoustophoretic experiment, the particle size *d*_p_ was 500 nm in diameter and the used RF voltage was 8.5 V with a frequency of 19.1 MHz. For this particle size, *κ *= 0.021, which guaranteed the streaming-dominated condition. In the acoustophoretic process, aggregation accompanies the strong rotational circulation. It can be observed that the particles aggregate rapidly into concentric arcs to form particle streams and then flow approximately along concentric circular paths. This corresponds to the encircling circulation pattern induced by Eckart streaming flow, which exert aggregation and circulation drag forces on the particles. Immediately afterwards, higher concentrations of particles agglomerate in several locations. We attribute this particle agglomeration effect to the trapping of particles by localized Rayleigh–Schlichting streaming vortices.

Based on a rough estimation of the experimental results, the particle velocity is of the order of 1.0 m/s. Compared with the maximum acoustic streaming velocity of 18.52 mm/s in the numerical results, the much lower acoustic streaming velocity obtained in the experiment is mainly attributed to the radiation loss and attenuation when SAWs pass through the viscoelastic PDMS cover layer before entering the microfluidic chamber. Nonetheless, the experimentally achieved acoustic streaming velocity is still significantly higher than previous results using plane-wave SAWs, so the dual focused SAW beams can lead to a strong rotational circulation flow in the chamber.

## 4. Conclusions

We investigated rotational circulation in a microfluidic chamber driven by dual focused SAWs. The dual focused SAWs were actuated at 19.1 MHz by two offset focused IDTs of radial pitch *p*_IDT_ = 200 μm. The excitation frequency and propagation behaviors of the focused SAW beams were numerically characterized using FE simulations, which demonstrated effective generation of highly focused SAWs with the designed IDTs. Accordingly, a full 3D modeling of the combined system of microfluidic chamber and SAW device was performed to simulate the first-order acoustic pressure field and second-order acoustic streaming field. The results showed that dual focused SAW beams can result in rotational acoustic pressure field and encircling acoustic streaming field in the chamber. Acoustophoretic experiments using submicron particles suspending in the microfluidic chamber verified the rotational circulation motion of the streaming flow, which is attributed to enhanced angular momentum flux injection and Eckart streaming effect through the dual focused SAW beams. Our results should be of importance in driving particle circulation and enhancing mass transfer in chamber embedded microfluidic channels, which may have promising applications in accelerating bioparticle or cell reactions and fusion, enhancing biochemical and electrochemical sensing, and efficient microfluidic mixing.

## Figures and Tables

**Table 1:** Geometric parameters of the designed device.

Geometrical Parameter	Symbol	Value
Thickness of LiNbO_3_ substrate Chamber diameter	*h*_LN_ *D*	500 μm 1000 μm
Channel height Main channel width First electrode radius of IDTs Electrode pair number of IDTs IDT pitch Coverage angle of IDTs Offset distance of IDTs	*h* *w* *R* *N_e_* *p*_IDT_ *θ* *_c_* *e*	200 μm 400 μm 1600 μm 20 200 μm 60° 500 μm

## Data Availability

The data that support the findings of this study are available within the article.

## Supplementary Materials

The following supporting information can be downloaded at: https://www.mdpi.com/article/10.3390/mi16020140/s1, Video S1: Supplementary Movie S2; Video S2: Supplementary Movie S2; Video S3: Supplementary Movie S3.
