# Supplementary figures and images for: Driving Rotational Circulation in a Microfluidic Chamber Using Dual Focused Surface-Acoustic-Wave Beams
Source: Micromachines (Basel). 2025 Jan 25;16(2):140. doi: 10.3390/mi16020140 (PMC11857384; doi:10.3390/mi16020140)

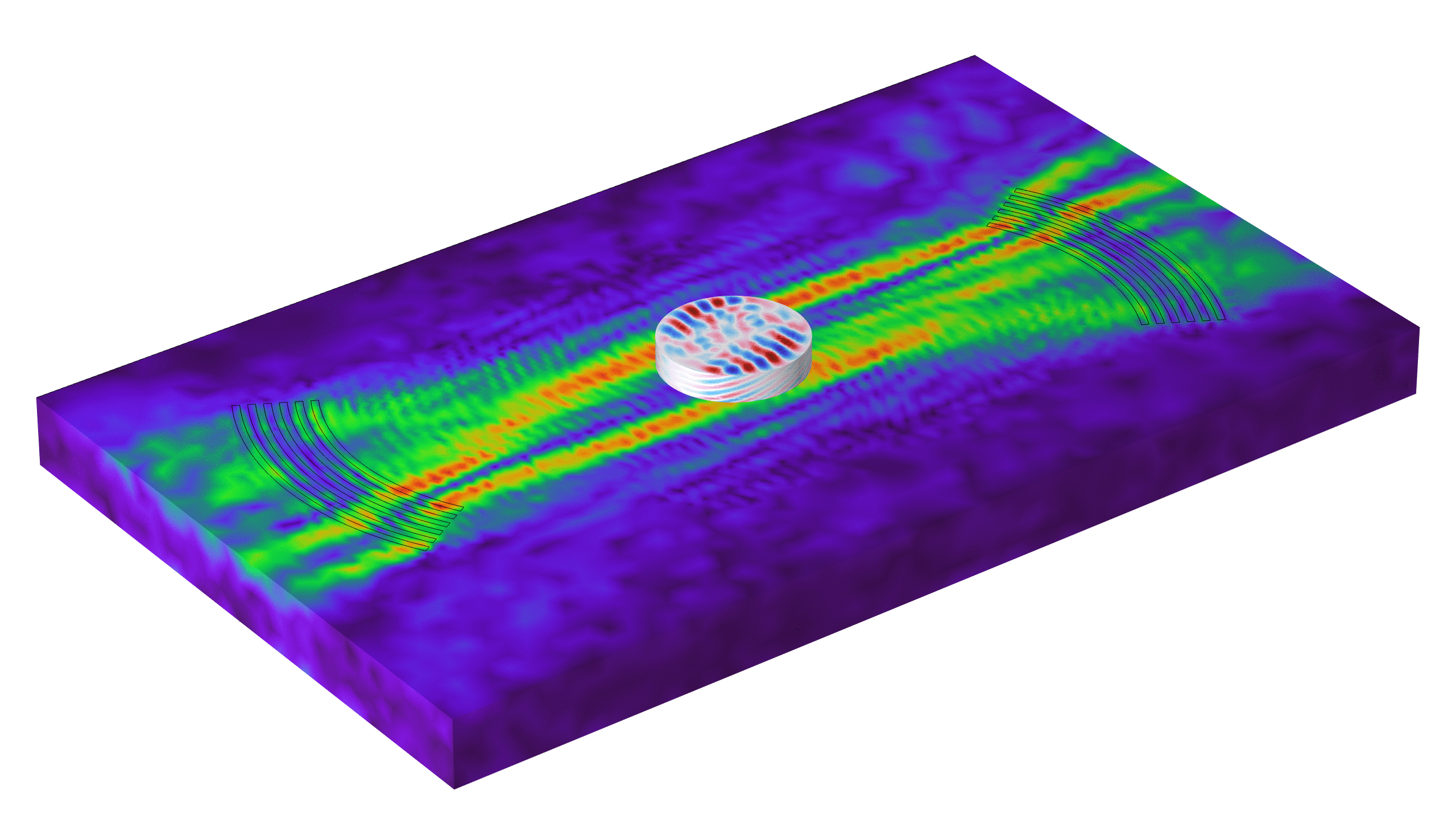

Supplement: Supplementary file 1 [file micromachines-16-00140-s001.zip › Supplementary files/Supplementary Movie S1.gif]

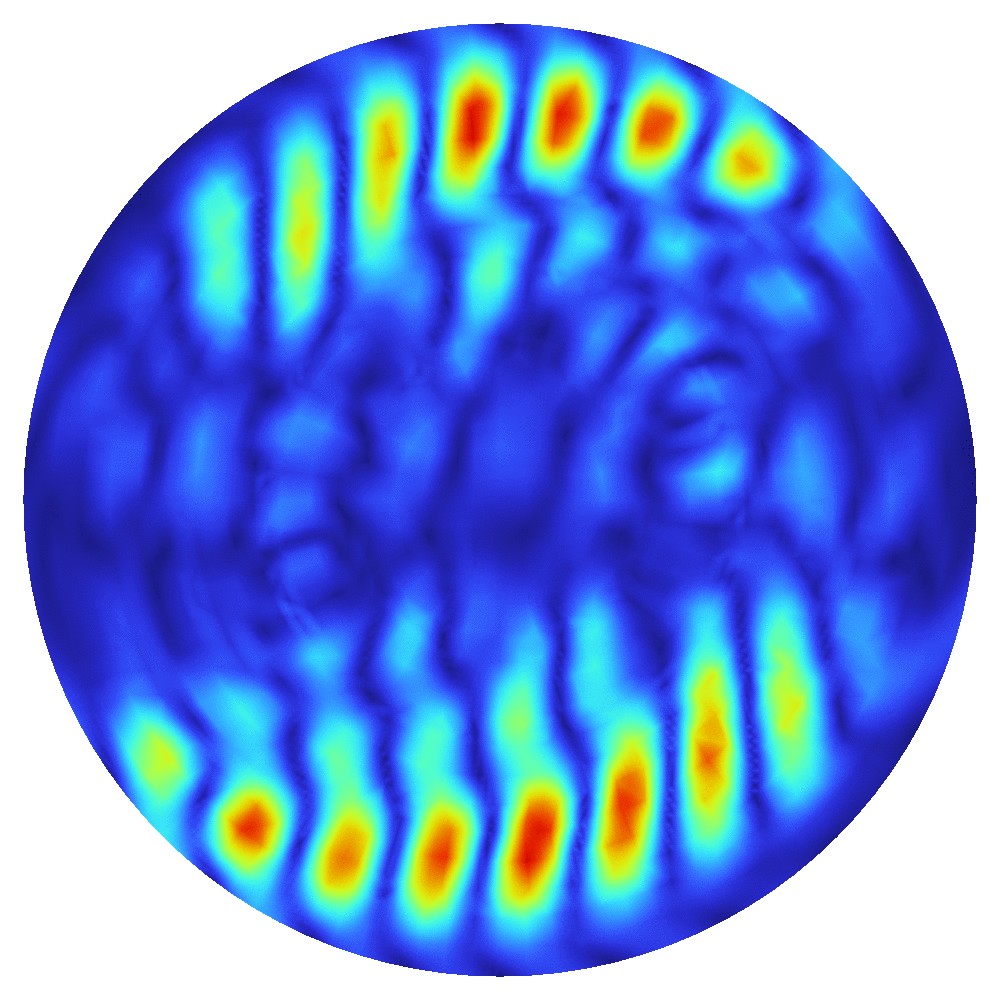

Supplement: Supplementary file 1 [file micromachines-16-00140-s001.zip › Supplementary files/Supplementary Movie S2.gif]
